# Molecular Interaction and Cellular Location of RecA and CheW Proteins in *Salmonella enterica* during SOS Response and Their Implication in Swarming

**DOI:** 10.3389/fmicb.2016.01560

**Published:** 2016-10-06

**Authors:** Oihane Irazoki, Jesús Aranda, Timo Zimmermann, Susana Campoy, Jordi Barbé

**Affiliations:** ^1^Department de Genètica i de Microbiologia, Universitat Autònoma de BarcelonaCerdanyola del Vallès, Spain; ^2^Advanced Light Microscopy Unit, Center for Genomic RegulationBarcelona, Spain

**Keywords:** SOS response, swarming, chemoreceptor polar arrays, chemosensory cluster assembly, RecA, CheW, 3D-STED

## Abstract

In addition to its role in DNA damage repair and recombination, the RecA protein, through its interaction with CheW, is involved in swarming motility, a form of flagella-dependent movement across surfaces. In order to better understand how SOS response modulates swarming, in this work the location of RecA and CheW proteins within the swarming cells has been studied by using super-resolution microscopy. Further, and after *in silico* docking studies, the specific RecA and CheW regions associated with the RecA-CheW interaction have also been confirmed by site-directed mutagenesis and immunoprecipitation techniques. Our results point out that the CheW distribution changes, from the cell poles to foci distributed in a helical pattern along the cell axis when SOS response is activated or RecA protein is overexpressed. In this situation, the CheW presents the same subcellular location as that of RecA, pointing out that the previously described RecA storage structures may be modulators of swarming motility. Data reported herein not only confirmed that the RecA-CheW pair is essential for swarming motility but it is directly involved in the CheW distribution change associated to SOS response activation. A model explaining not only the mechanism by which DNA damage modulates swarming but also how both the lack and the excess of RecA protein impair this motility is proposed.

## Introduction

RecA is a multifunctional protein present in almost all members of the Bacteria domain (Eisen, 1995). In the presence of single-stranded DNA (ssDNA), generated, for instance, by direct or indirect DNA damage, RecA becomes activated (RecA^∗^) (Sassanfar and Roberts, 1990; Michel, 2005) acquiring co-protease activity that prompts auto-cleavage of the LexA repressor which governs the SOS response (Little, 1991). LexA cleavage triggers not only the expression of *recA* itself but also that of other SOS genes, mostly those involved in DNA recombination and repair (Courcelle et al., 2001). Further, it has been described that RecA is associated with the cell membrane forming foci often located at the cell poles that are redistributed along the cell in response to DNA damage (Renzette et al., 2005; Lesterlin et al., 2014; Rajendram et al., 2015). RecA, however, aside from its role in DNA damage repair and as a DNA damage sensor, has been directly related to swarming motility (Gómez-Gómez et al., 2007; Medina-Ruiz et al., 2010), through its interaction with the CheW protein (Mayola et al., 2014; Irazoki et al., 2016).

Swarming motility is the rapid, organized multicellular translocation of bacteria across a moist surface. It is powered by rotating flagella (Henrichsen, 1972) and is widely distributed through the Bacteria Domain (Harshey, 1994). Swarming is associated with elevated resistance to multiple antibiotics (Ottemann and Miller, 1997; Kim and Surette, 2003; Kim et al., 2003; Wang et al., 2004; Overhage et al., 2008; Katribe et al., 2009) and is essential for bacterial colonization of host surfaces (Nakajima et al., 2008; Barak et al., 2009; Katribe et al., 2009). Like other components of the chemotaxis pathway, the CheW protein plays a key role in swarming ability (Burkart et al., 1998; Mariconda et al., 2006). As the chemoreceptor adaptor, CheW couples the transmembrane methyl-accepting chemoreceptor protein trimers of dimers (MCPs) to CheA, a histidine kinase that transfers the signal to the CheY response regulator, which acts on the flagellar motor by switching flagellar rotation according to the stimuli detected by the MCPs (Boukhvalova et al., 2002; Sourjik and Wingreen, 2012). To avoid saturation of the sensory system, the chemoreceptor signal is reset by the activity of a methyltransferase (CheR) and a methylesterase (CheB), both of which are located in the vicinity of the chemoreceptors and which restore pre-stimulus activity through reversible covalent modification of the MCPs (Sourjik and Wingreen, 2012).

These signaling complexes pack together to form large chemoreceptor arrays, ranging from a few to 1000s of proteins and located at the cell poles. By acting as antennae, they amplify the signal generated in response to slight changes in the concentrations of attractants or repellents detected by MCPs (Briegel et al., 2012, 2014a; Sourjik and Wingreen, 2012; Cassidy et al., 2015). The chemoreceptor array assembly has been the focus of several studies (Shiomi et al., 2006; Thiem and Sourjik, 2008; Jones and Armitage, 2015). The newly synthesized signaling complexes are distributed in a helical fashion at the cell membrane via their association with cytoskeletal proteins such as MreB or the Sec secretion system (Shiomi et al., 2006; Oh et al., 2014). Then, by stochastic self-assembly (Greenfield et al., 2009) or by an active process (Shiomi et al., 2006), these complexes form large clusters by joining existing arrays or by the formation of new nucleation centers. Stabilization of these clusters is a function of both the cell membrane curvature in the polar region (Oh et al., 2014) and the presence of CheA and CheW (Shiomi et al., 2005). These proteins are directly involved in the stabilization of these clusters, as they interact to form structural core linkers [CheW-CheA_2_-CheW] across the cytoplasmic domain of MCPs, thereby clustering the chemoreceptors into hexagonal rings. The assembled array may thus contain dozens to 100s of hexagons (Briegel et al., 2014b; Cassidy et al., 2015). Within the hexagons is a CheW ring that couples neighboring chemoreceptors and strengthens the stability of the chemosensory array (Cassidy et al., 2015).

The presence of polar chemoreceptor arrays is essential for swarming motility in soft swarmers, such as *Escherichia coli* and *Salmonella enterica* (Cardozo et al., 2010; Santos et al., 2014). In the latter bacterium, an alteration in the balance of RecA/CheW impairs chemoreceptor cluster assembly and thus modulates bacterial swarming motility (Cardozo et al., 2010; Medina-Ruiz et al., 2010; Irazoki et al., 2016). The overexpression of RecA, without its activation, is sufficient to abolish swarming (Irazoki et al., 2016). Thus, by using RecA as a sensor mechanism, *S. enterica* cells can adapt their surface motility in response to the presence of direct or indirect DNA-damaging agents, by sensing these compounds through SOS system induction (Irazoki et al., 2016).

Although, RecA is known to interact with CheW (Mayola et al., 2014), where the interaction occurs within the cell and its nature are poorly understood. In an attempt to answer these questions and to better understand how the SOS response modulates swarming, in this work we have determined the regions involved in RecA and CheW interaction and the location of these proteins within SOS response induced*-S. enterica* swarming cells as well as RecA-CheW interaction relationship with swarming motility.

## Materials and Methods

### Bacterial Strains and Growth Conditions

All bacterial strains and vectors used in this work are indicated in **Table 1**. Except when indicated, all strains were grown at 37°C in Luria-Bertani (LB) broth or on LB plates, supplemented, when necessary, with ampicillin (100 μg/mL), chloramphenicol (34 μg/mL), and/or kanamycin (10 μg/mL).

**Table 1:** Bacterial strains and plasmids used in this work.

Strains	Relevant characteristic(s)	Source or reference
DH5α	*E. coli supE4 ΔlacU169 (ϕ80 ΔlacZ ΔM15) hsdR17, recA1, endA1, gyrA96, thi-1, relA1*	Clontech
ATCC14028	*S. enterica* Typhimurium wild-type	ATCC
UA1915	*S. enterica* Typhimurium *ΔrecA ΔcheW*	Mayola et al., 2014
UA1916	*S. enterica* Typhimurium *cheW*::FLAG	Irazoki et al., 2016
UA1941	*S. enterica* Typhimurium *cheW*::FLAG *ΔrecA*	This work
UA1942	*S. enterica* Typhimurium *cheW*::FLAG *recA*::HA	This work
UA1943	*S. enterica* Typhimurium *cheW*::FLAG *recA*::HA pNASΩ*cheR*::eYFP	This work
MC1061	F^-^λ^-^Δ*(ara-leu)7697* [*araD139*]B/r Δ*(codB-lacI)3 galK16 galE15* e14 *mcrA0 relA1 rpsL150*(Str^R^) *spoT1 mcrB1 hsdR2*(*r*^-^*m*^+^)	CGSC

**Plasmids**		

pKOBEGA	Vector containing the /g/g/g Red recombinase system, Amp^r^, temperature sensitive OriV	Chaveroche et al., 2000
pKD3	Vector carrying FRT-Cm construction, Amp^R^, Cm^R^	Datsenko and Wanner, 2000
pCP20	Vector carrying FLP system, OriVts, Amp^R^	Datsenko and Wanner, 2000
pGEX-4T-1	Expression vector carrying the P*tac* IPTG- inducible promoter and the *lacI^q^* gene; GST fusion tag, Amp^R^	Amersham Biosciences
pKO3	Vector for homologous recombination. temperature sensitive OriV, *sacB*, Cm^R^	Link and Phillips, 1997
pUA1108	pGEX 4T-1 derivative plasmid carrying only the P*tac* promoter and the *lacI^q^* gene; used as overexpression vector for *recA* and *cheW* wild-type and mutant derivative genes, Amp^R^	Mayola et al., 2014
pUA1135	pUA1108 derivative containing the native *S. enterica* Typhimurium *recA::HA* gene under the control of the P*tac* promoter, Amp^R^.	This work
pUA1131	pUA1108 derivative containing the native *S. enterica* Typhimurium *cheW::FLAG* gene under the control of the P*tac* promoter, Amp^R^.	Mayola et al., 2014
pUA1136	pKO3 derivative carrying *recA::HA* fusion, Cm^R^	This work


### Stimulated Emission Depletion (STED) Microscopy

Fluorescent immunolabeling was carried out as described (Buddelmeijer et al., 2013), with a few modifications. All samples were obtained from the edge of the corresponding swarming plates as previously described (Kim and Surette, 2005). The cells were grown, as described below for swarming assays, on LB-swarming plates [1% tryptone, 0.5% yeast extract, 0.5% NaCl, 0.5% D-(+)-glucose, and 0.5% agar] supplemented when needed with 0.08 mitomycin C/mL or 30 μM of IPTG. After 14 h of incubation at 37°C, the cells were suspended in 1 mL of ice-cold tethering buffer (10 mM potassium-phosphate pH 7, 67 mM NaCl, 10 mM Na-lactate, 0.1 mM EDTA, and 0.001 mM L-methionine) by gently tilting the plates back and forth and harvested by 15 min of low-speed centrifugation (5000 *g*). With this method, migrating cells were easily lifted off the surface, whereas the vast majority of cells in the middle of the plates remained intact on the surface. Non-swarming colonies were recovered using the same method but with 0.5 mL of cold tethering buffer.

Then, the cells were permeabilized by two subsequent treatments with 0.1% Triton X-100 and freshly prepared PBS-lysozyme-EDTA buffer (1× PBS, 100 μg lysozyme/mL and 5 mM EDTA), each for 1 h at room temperature. Then they were incubated in 0.5% blocking reagent (Sigma-Aldrich) at 37°C for 30 min with shaking. After, cells were then centrifuged at 4,500 *g* for 5 min, re-suspended in 100 μL of the appropriate primary antibody (diluted 1:20), and incubated overnight at 37°C. After three washes in wash buffer (1× PBS, 0.05% Tween20), the cells were recovered by centrifugation at 4,500 *g*, re-suspended in 100 μL of the secondary antibody (diluted 1:100), and incubated at 37°C for 2 h without shaking. Finally, after three washing steps with wash buffer, the labeled cells were resuspended in 1× PBS and placed on 35-mm poly-L-lysine pre-coated coverslips using Mowiol-DABCO mounting medium (1× PBS, 2.5% DABCO, 25% Mowiol, and 1× glycerol). The samples were air-dried and then examined under an AxioImager M2 microscope (Carl Zeiss Microscopy) equipped with the appropriate filter set (for the green channel the GFP (Zeiss filter set 38) and for the red Rhod (Zeiss filter set 20) to ensure that at least 90% of cells were correctly permeabilized and immunolabeled. Afterward, at least 300 double marked cells were visually inspected in each sample and the presence and type of clusters as well as RecA distribution were analyzed. Each experiment was performed in triplicate using independent cultures; a minimum of 900 cells from each studied *S. enterica* strain or condition were therefore examined. In all cases, at least the 70% of cells present the same RecA and CheW distribution profile.

Afterward, super-resolution images of the previously analyzed samples were taken on a Leica TCS SP8 STED3X microscope (Leica Microsystems) using a highly corrected 1.4 NA 100x Plan Apo objective specified for STED imaging. Imaging was done using the lateral resolution improvement lightpath (z-STED set to zero). The fluorophore labels were emission depleted using a 660 nm continuous wave (CW) laser for the stimulated emission effect and time-gating (rejection of early emission events) to further increase the resolution. Data were acquired in the form of two channel z-stacks for subsequent deconvolution and rendering.

**FIGURE 1:**
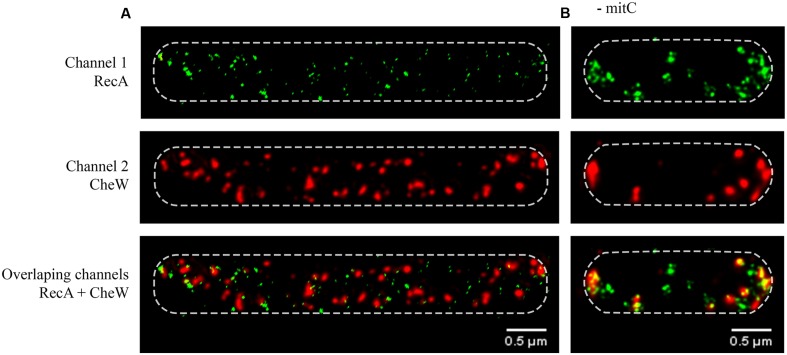
**Representative STED (Stimulated Emission Depletion) images of the subcellular locations of RecA and CheW in *Salmonella enterica* wild-type swarming cells **(A)** in the presence of mitomycin C (0.08 μg/mL) or **(B)** in the absence of this SOS inducer.** The RecA and CheW proteins were labeled with Alexa Fluor^®^ A488 (channel 1) and Alexa Fluor^®^ A568 (channel 2), respectively. For all images, each channel is shown both individually and overlapped. In all cases, the maximum intensity projection images of the obtained z-stacks are shown.

**FIGURE 2:**
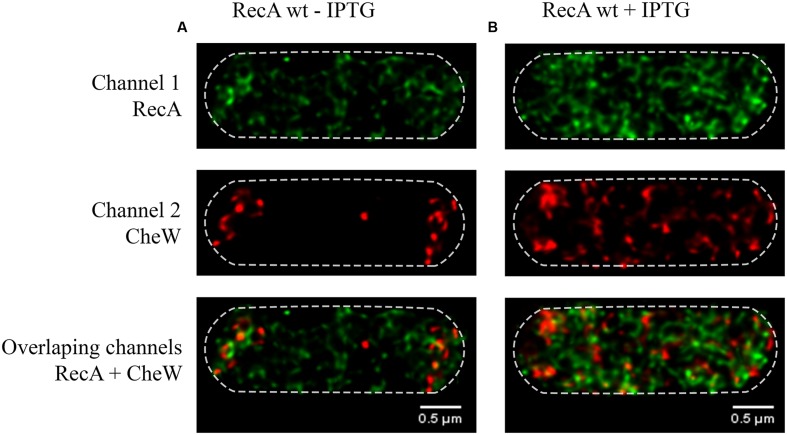
**Representative STED images of the subcellular locations of RecA and CheW in the *S. enterica ΔrecA* overexpressing strain.** **(A)** The effect of basal expression of wild-type RecA in the absence of IPTG. **(B)** Addition of the inducer (30 μM of IPTG) yielded the overexpression of wild-type RecA and the change of the CheW distribution within the cell. It is worth noting that the basal expression of wild-type RecA recovers swarming ability. RecA and CheW proteins were labeled with Alexa Fluor^®^ A488 (channel 1) and Alexa Fluor^®^ A568 (channel 2), respectively. In the images, each channel is shown individually and overlapped. In all cases, the maximum intensity projection images of the obtained z-stacks are shown.

For deconvolution of the z-stacks the STED module of the Huygens software package (Scientific Volume Imaging, SVI) was used. Images were analyzed using ImageJ software (National Institutes of Health). In all cases, images of 10 different randomly chosen cells were obtained for each sample. As each experiment was performed in triplicate, a total of 30 cells from each studied strain or condition were therefore examined. The images presented in **Figures 1**, **2**, and **7** are representative of the entire image set. All images shown in the Figures present the same contrast settings.

### *In silico* Protein–Protein Interaction Docking

Simple protein–protein docking was conducted using the ClusPro server (Comeau et al., 2004a) to generate an *in silico* model for the RecA-CheW protein complex. The available resolved structures of the *E. coli* RecA (PDB: 2REB) and CheW (PDB: 2HO9) proteins were used to run the analyses. The resultant model was presumed to be reliable also for *S. enterica* as the reported BLAST identity between *E. coli* K-12 and *S. enterica* sv. Typhimurium ATCC14028 proteins is 97 and 92% for RecA and CheW, respectively.

The protein–protein docking assay was carried out in duplicate, selecting RecA as the receptor and CheW as the ligand, and *vice versa*. The protein structures and the obtained *in silico* models were visualized and analyzed using PyMOL software (Schrödinger, 2010).

### Co-immunoprecipitation Assay

The cell lysates were obtained as previously described (Irazoki et al., 2016). Cultures of *S. enterica ΔrecA ΔcheW* harboring the plasmids encoding the corresponding tagged proteins were used, and the gene overexpression was induced by the addition of 1 mM IPTG. As a control, cell lysates of *S. enterica ΔrecA ΔcheW* containing the pUA1108 overexpression vector (Mayola et al., 2014) were processed according to the same procedure.

The immunoprecipitation assays were performed using Pure Proteome Protein A magnetic beads (Millipore) coated with either mouse anti-FLAG IgG (Sigma-Aldrich) or mouse anti-HA IgG (Sigma-Aldrich) monoclonal primary antibodies, following the manufacturer’s instructions. Cell lysates were mixed at a molecular ratio of 1:1 and incubated at 30°C for 1 h without shaking to allow protein–protein interaction.

As a final step, the samples were separated by SDS-PAGE on a 15% polyacrylamide gel and analyzed by Western blotting using a horseradish-peroxidase (HRP)-coupled anti-mouse antibody (Acris). The membranes were developed using a HRP chemoluminiscent substrate (Luminata ForteTM Western HRP substrate, Millipore) following the manufacturer’s instructions. The membranes were imaged using a ChemiDocTM XRS+ system (Bio-Rad).

### Construction of RecA and CheW Tagged Proteins

CheW::FLAG- and RecA::HA-carrying plasmids were constructed by PCR-amplifying the *recA* and *cheW* genes using the appropriate oligonucleotide pairs (**Supplementary Table S1**). In both cases, the corresponding tag sequence was included at the 3′ end of the gene, such that the tag was placed at the C-terminus of the protein. A 3×Gly linker between the tag and the gene sequence was also added. The same strategy was used to obtain the *recA* and *cheW* tagged mutant derivatives. In this case, the oligonucleotides included the suitable point mutation (**Supplementary Table S1**).

All PCR products were digested and cloned into pUA1108 overexpression vector (Mayola et al., 2014) under the control of the IPTG-inducible Ptac promoter. These plasmids were transformed into *E. coli* DH5α and confirmed by sequencing. When needed, the plasmids containing the tagged proteins were transformed into the corresponding *S. enterica* strains with the appropriate genetic backgrounds. The selected transformants were confirmed again by PCR and sequencing.

The *cheW::*FLAG *recA::*HA double-tagged strain was constructed as described previously (Irazoki et al., 2016), using the pKO3 plasmid (Latasa et al., 2012). The *recA::*HA construct was obtained by PCR overlap-extension (which added the epitope YPYDVPDYA to the RecA protein), cloned into the pKO3 vector, and introduced into the previously constructed *S. enterica cheW::*FLAG strain (Irazoki et al., 2016). The *S. enterica ΔrecA cheW::*FLAG strain was constructed by one-step PCR gene replacement as described previously (Datsenko and Wanner, 2000; Irazoki et al., 2016) using the *S. enterica cheW::*FLAG strain as a recipient strain. In all cases, it was confirmed that neither the FLAG nor the HA tag insertion affected the surface motility of the tagged strains.

### Swarming Motility and Biofilm Assays

Swarming assays were carried out as described previously (Gómez-Gómez et al., 2007; Mayola et al., 2014; Irazoki et al., 2016). In short, a single colony was picked from bacterial strains grown on LB plates at 37°C and inoculated in the center of a freshly prepared LB swarming plate [1% tryptone, 0.5% yeast extract, 0.5% NaCl, 0.5% D-(+)-glucose, and 0.5% agar] using a sterile toothpick and avoiding medium penetration. The plates were supplemented with IPTG (10 or 30 μM) or mitomycin C (0.08 μg/mL) as needed, incubated overnight at 37°C, and then imaged using a ChemiDocTM XRS+ system (Bio-Rad).

The phenotypic assays for biofilm formation were performed as previously described (Latasa et al., 2012). After 96 h of incubation at 25°C without agitation, the biofilm formed in standing LB broth was visualized as a floating pellicle at the air–broth interface that totally blocked the surface of the culture and could not be dispersed by shaking.

### Recombinase Activity Assay

To determine the recombination efficiency of the *S. enterica* strains carrying overexpression vectors containing the *recA* tagged mutant derivatives, the P22 transduction frequency of the strain was compared with that of the same strain but carrying an overexpression vector with wild-type tagged *recA*; the method was described previously (McGrew and Knight, 2003). The transduction experiments were performed as described elsewhere (Campoy et al., 2002). The recombination efficiency was calculated as the number of transductants relative to the initial recipient cell concentration. The relative recombination frequency was the recombination efficiency (%) of each overexpressing strain with respect to the strain overexpressing wild-type *recA*.

### ELISA for RecA and CheW Quantification

RecA and CheW quantification was performed by ELISA as described before (Irazoki et al., 2016). All samples recovered from the colony edge were resuspended in sonication buffer (PBS 1×, cOmplete mini EDTA-free tablets, pH 7.3) and sonicated (2x 30-s pulses and 20% amplitude, Digital sonifierR 450, Branson) obtaining the whole cell lysates. After centrifugation (12000 *g* for 10 min), the supernatants were recovered and the total protein concentration of each sample was quantified according to the Bradford method using the protein reagent DyeR (BioRad) and a bovine serum albumin standard curve (range: 1.5–200 μg/mL).

The RecA and CheW::FLAG proteins used in the standard quantification curves were obtained as previously described (Irazoki et al., 2016). The RecA::HA and CheW::FLAG proteins were quantified by ELISA as described (Mayola et al., 2014) using anti-RecA (monoclonal antibody to ARM193 RecA clone, MBL) and anti-FLAG (monoclonal antibody to DYKDDDDK epitope Tag, Acris) mouse IgG. The secondary antibody was an anti-mouse-IgG horseradish-peroxidase-conjugated antibody [polyclonal antibody to mouse IgG (HEL)-HRP, Acris]. The BD OptEIA TMB substrate reagent set (BD Biosciences), prepared following the manufacturer’s instructions, was used as the developing solution. Plate measurements were made at 650 nm using a multiplate reader (Sunrise, Tecan).

## Results

### Subcellular Localization of RecA and CheW Proteins in Swarming Cells during SOS Induction

The location of RecA and CheW proteins within SOS response activated*-S. enterica* swarming cells was analyzed by using 3D-stimulated emission depletion microscopy (3D-STED), a super-resolution fluorescence imaging technique that increases axial resolution by up to 20–40 nm in biological samples (Han and Ha, 2015). Thus, a *S. enterica cheW::FLAG recA::HA* strain was constructed and the appropriate antibodies were used to locate these proteins inside the swarming cells in the presence of a SOS response inducer.

As it is shown in **Figure 1**, besides the expected cell filamentation due to the induction of the SOS response by mitomycin C, the SOS inducer treatment gives rise to a dramatic change in the subcellular location of both RecA and CheW within cells cultured on swarming plates containing sub-lethal concentration of mitomycin C (**Figure 1A**). In agreement with *E. coli* cells grown in liquid medium under non-DNA-damaging conditions (Greenfield et al., 2009), in the non-mitomycin C-treated *S. enterica* swarming cells, the CheW protein was majorly located at the cell poles (**Figure 1B**). This CheW location is the same than that previously described for chemoreceptor polar arrays and accordingly more than 70% of cells presented chemoreceptor polar clusters in these conditions (Kentner et al., 2006; Greenfield et al., 2009; Cardozo et al., 2010; Mayola et al., 2014; Santos et al., 2014).

Nevertheless, the SOS response induction prompts a change in the CheW distribution, which instead of being at the cell poles was indeed organized in smaller foci distributed in a spiral-like configuration along the cell membrane (**Figure 1A**). Further, and in agreement with previous data about cluster assembly under SOS induction (Irazoki et al., 2016), this CheW distribution was present in more than 70% of analyzed cells. Likewise, the CheW foci resembled the distribution and organization of RecA (**Figure 1A**). Under this DNA-damaging conditions, the SOS system induction gives rise not only an increase in the RecA concentration (Irazoki et al., 2016) but also to a higher amount of cellular RecA aggregates (**Figure 1A**). After SOS induction, the RecA foci seemed to be smaller and were distributed not only at the cell poles but also along the filamented cell axis, assuming a helical configuration just underneath the bacterial wall (**Figure 1A**). These observations were in agreement with the previously reported changes in the location of RecA in liquid cultures of *E. coli* cells growing in DNA-damaging conditions and the described reduction of RecA storage structures at cell poles (Renzette et al., 2005; Lesterlin et al., 2014; Rajendram et al., 2015). Nevertheless, in our experimental settings, using sub-lethal concentration of mitomycin C, RecA was not distributed forming bundles as those described for *E. coli* (Lesterlin et al., 2014), that were only observed when *S. enterica* was grown in liquid cultures adding higher amount of the SOS inducer (data not shown).

To rule out an indirect effect of either DNA damage or SOS-dependent filamentation on the CheW distribution and to determine whether RecA activation plays a significant role in the distribution of its partner protein, *recA* was overexpressed under non-DNA-damaging conditions and the locations of the CheW and RecA tagged proteins were examined.

In this experiment, *S. enterica Δ*recA *cheW::FLAG* strain carrying the pUA1135 vector, the pUA1108 overexpression vector containing the *recA::HA* gene under the control of an IPTG-inducible promoter was used (**Table 1**). Induction was achieved by adding IPTG to the swarming plates. The basal expression of the wild-type *recA* carried in this plasmid is enough to recover both swarming ability and the polar-clustered CheW arrangement (**Figure 2A**). In agreement, more than 70% of cells present polar chemoreceptor arrays. Following the addition of IPTG to the swarming plates, the increase in RecA was accompanied by a helical distribution of CheW along the cell axis (**Figure 2B**), as occurs following activation of the SOS response (**Figure 1B**). And, as expected, about 70% of cells did not present polar chemoreceptor clusters.

These findings indicate that neither the cell filamentation, the activated RecA protein nor DNA damage is required to modify the subcellular location of CheW.

### *In silico* Prediction of the RecA-CheW Interaction

To further determine how does the RecA-CheW interaction occurs, the RecA and CheW residues associated with this interaction were identified by using an *in silico* model for RecA-CheW complex-formation in which simple protein–protein docking was conducted using the resolved ternary structures *E. coli* RecA (PDB: 2REB) and CheW (PDB: 2HO9).

RecA has three major functional domains. The amino domain contains a large α-helix and short β-strand that are implicated in the formation of the RecA polymer. The central domain (consisting primarily of a twisted β-sheet with eight β-strands bound by eight α-helices) is involved in DNA and ATP binding. The carboxyl domain is made up of three α-helices and three β-strands that facilitate interfilamentous associations (Story et al., 1992). On the other hand, the folded CheW has a SH3-like regulatory domain and two intertwined five-stranded β-barrels, designated subdomains 1 and 2 (Li et al., 2013).

As little is known about the forces guiding protein-complex formation, balanced-coefficient docking models were considered to be the most accurate for the analysis of the RecA-CheW interaction (Comeau et al., 2004b). Ten of the highest-scoring models were analyzed individually for each combination of RecA receptor protein and CheW ligand, and *vice versa*. Although, the spatial arrangement was not exactly the same in each paired combination, the results allowed the putative interacting regions of each protein to be identified, as they were those that were repeated in all models.

**FIGURE 3:**
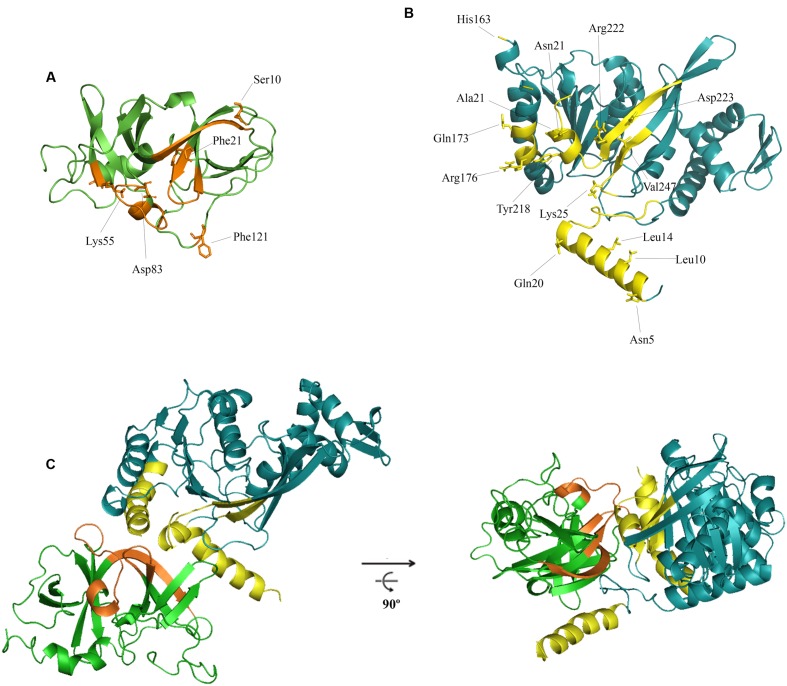
***In silico* model for the interaction of RecA and CheW proteins.** The predicted ternary structures of *S. enterica* CheW **(A)** and RecA **(B)** proteins are shown. The putative interface of RecA and CheW involved in the reciprocal interaction of the two proteins is highlighted in yellow and orange, respectively. The residues selected for site-directed mutagenesis and their locations are also indicated. **(C)** Ribbon diagrams of one of the highest-scoring models generated for RecA-CheW pair formation analyzed in this study. The two views of the interaction are rotated 90° about the *x*-axis.

**Figure 3** shows the residues of the folded RecA that putatively participate in the interaction with CheW. These were predicted to be located in the amino-terminal and central domains (in α1, α10, α11, β8, and β9) whereas the presumed CheW regions were located in both subdomains, specifically, in the β1 and β4 (subdomain 1) and the T4, β8, and B6 regions (subdomain 2).

### Mutational Analysis the RecA-CheW Pair Formation

To corroborate the interaction interfaces identified *in silico*, site-directed mutagenesis was used to construct several mutant derivatives for each protein in which the relevant residues were affected. In all cases, the corresponding *recA* and *cheW* gene mutants constructed *in vitro* were HA- and FLAG-tagged, respectively, and cloned into the overexpression vector (pUA1108) under the control of an IPTG-inducible promoter.

Fourteen RecA and five CheW residues were selected based on their potential roles in RecA-CheW pair formation (**Tables 2** and **3**) as well as their reactivity and exposure on the corresponding protein surface (**Figure 3**). With the exception of the A214V RecA mutant, in which the Ala residue was changed to a Val, all other selected residues were converted to an Ala (**Tables 2** and **3**), as this aliphatic amino acid is considered to be non-reactive (Cunningham and Wells, 1989). The effect of each substitution on the RecA-CheW interaction was determined *in vitro* and *in vivo* by co-immunoprecipitation and swarming inhibition assays, respectively.

**Table 2:** *In vitro* interaction of RecA mutant derivatives with wild-type CheW protein.

RecA protein^a^	Secondary structure region containing the mutated residue	Interaction with wild-type CheW^b^	Swarming inhibition by RecA overexpression^c^
Wild-type	NA^d^	+	+
L10A	Helix α1	+	+
L14A		+	+
Q20A		-	-
H163A	NR^d^	+	+
Q173A	Helix α10	+	+
R176A		-	-
N213A	Helix α11	+	+
A214V		+	+
K216A		+	+
Y218A		+	+
R222A	Strand β8	-	-
D224A		+	+
V247A	Strand β9	+	+
K250A	NR^d^	-	-


*^a^The mutated residue and the substitution of each tagged mutant derivative are indicated.*

*^b^Results of co-immunoprecipitation assays using each RecA derivative and wild-type CheW. (+) and (-) indicate the maintenance or abolishment of the RecA-CheW complex, respectively.*

*^c^Results of the *in vivo* swarming assays in which each RecA derivative was overexpressed in the *S. enterica* wild-type strain. (+) overexpression generates a non-swarming phenotype; (-) swarming was observed despite overexpression of the mutant protein.*

*^d^NA, not applied; NR, non-resolved secondary structure.*

**Table 3:** *In vitro* interaction of CheW mutant derivatives with wild-type RecA.

CheW protein^a^	Secondary structure region containing the mutated residue	Interaction with wild-type RecA^b^	Swarming inhibition by CheW overexpression^c^
Wild-type	NA^d^	+	+
F21A	Strand β1	-	-
K55A	Strand β4	-	-
D83A	Turn-6	-	-
S109A	Strand β8	+	+
F121A	Bend-6	-	-


*^a^The mutated residue and the substitution of each tagged mutant derivative are indicated.*

*^b^Results of the co-immunoprecipitation assays between each CheW derivative and the wild-type RecA protein. (+) and (-) indicate the maintenance or the abolishment of RecA-CheW, respectively.*

*^c^Results of the *in vivo* swarming assays by overexpressing each CheW derivative in the *S. enterica* wild-type strain. (+) overexpression generates a non-swarming phenotype; (-) swarming was observed despite overexpression of the mutant protein.*

*^d^NA, not applied.*

**FIGURE 4:**
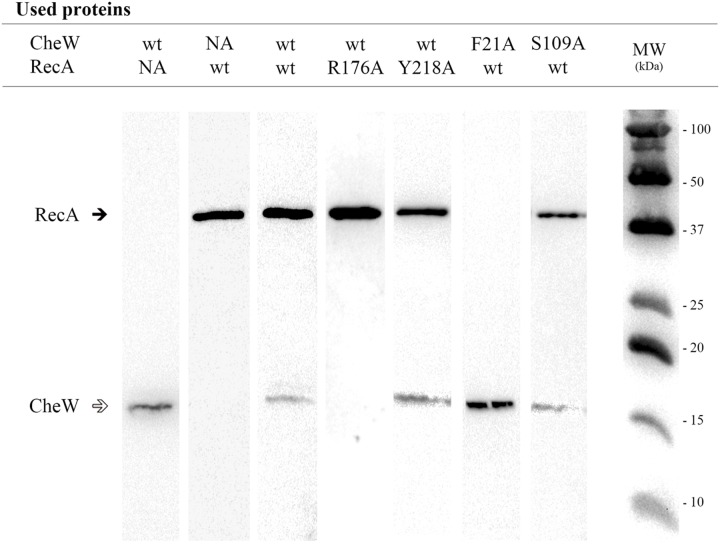
**Co-immunoprecipitation assays of RecA and CheW *S. enterica* mutant derivatives.** Representative images of the co-immunoprecipitation of mutant derivatives that allow (Y218A RecA::HA or S109A CheW::FLAG) or impair (R176A RecA::HA or F21A CheW::FLAG) the interaction of RecA-CheW are shown. Each lane contains a mixture of cell lysates prepared from the overexpressed mutant derivative and the wild-type (wt) tagged protein. As controls, mixtures containing either wild-type (wt) RecA or CheW or both were included. All experiments were done at least in triplicate; in all cases, the results for all mutant derivatives were exactly the same as those shown in the figure. Black and white arrows indicate RecA and CheW protein bands, respectively. NA, non-added; MW, molecular mass marker.

For the *in vitro* co-immunoprecipitation assays, each RecA::HA mutant protein was mixed with wild-type CheW::FLAG; anti-HA-antibody coated beads were used to hijack the proteins. The CheW::FLAG mutated derivatives were mixed with the RecA::HA wild-type protein and hijacked using anti-FLAG-antibody coated beads. Previous assays confirmed the ability of RecA::HA to pull down CheW::FLAG and *vice versa* (Mayola et al., 2014). It was therefore expected that if the mutated residue altered the RecA-CheW interaction, the antibody would pull down only the corresponding tagged mutant protein and would not co-immunoprecipitate both tagged proteins (**Figure 4**). The results showed that, among the mutants tested, only four RecA (Q20A, R222A, K250A, and R176A) and four CheW (F21A, D83A, K55A, and F121A) mutants impaired the RecA-CheW interaction (**Tables 2** and **3**). These results corroborated the *in silico* docking predictions and pointed out that some residues from α1, α10, and β8 regions of RecA and from the β1, β4, T6, and B6 regions of CheW participate in the interaction between the two proteins.

**FIGURE 5:**
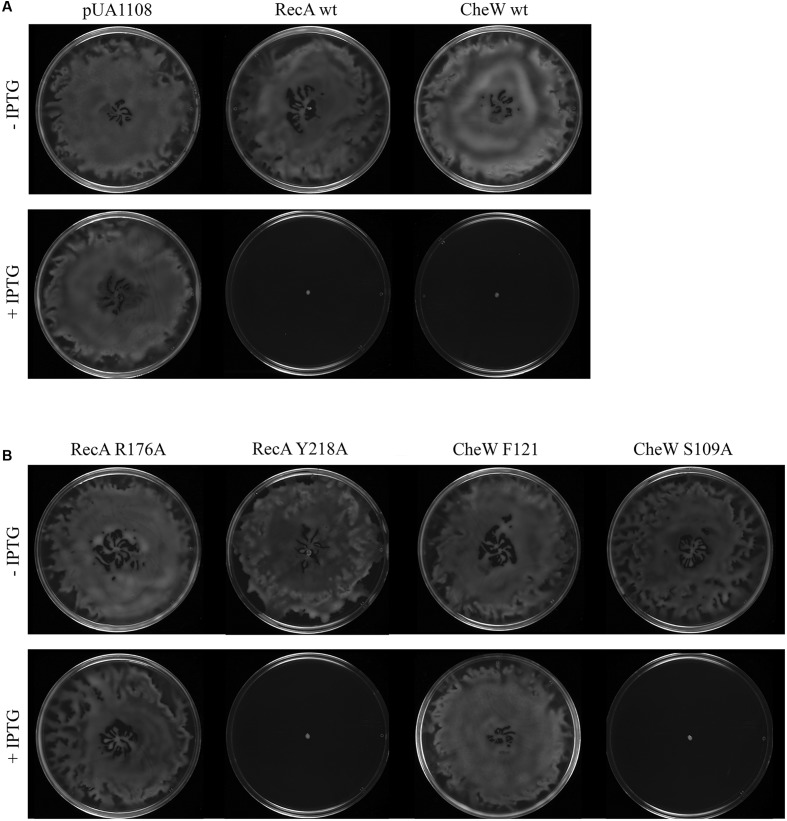
**(A)** Representative images of the *in vivo* swarming ability of the *S. enterica* wild-type (wt) strain overexpressing either RecA wt or CheW wt proteins or containing only the overexpression vector (pUA1108). **(B)** *In vivo* swarming assay of RecA and CheW mutant derivatives. Representative images of the swarming ability of *S. enterica* wt strain containing the overexpression vectors encoding RecA or CheW mutant derivatives that allow or impair RecA-CheW coupling are presented. The cells were cultured in the presence (+) or absence (-) of 30 μM IPTG. Swarming was inhibited in the presence of IPTG only in mutants that maintained the RecA-CheW interaction (RecA Y218A and CheW S109A). When the interaction of the two proteins was abolished (using RecA R176A and CheW F21A mutant strains), swarming was not affected when IPTG was added. Each experiment was performed at least in triplicate. The same results were observed for all of the mutant derivatives tested.

To determine the contribution of these eight residues to swarming motility, *in vivo* swarming assays were carried out using the constructed mutants. As it has been previously mentioned, the overexpression of either RecA or CheW inhibits swarming (Cardozo et al., 2010; Medina-Ruiz et al., 2010; Irazoki et al., 2016; **Figure 5A**). In these swarming assays the effect on swarming of RecA and CheW mutant derivative overexpression in wild-type cells was determined. For this reason, all the vectors overexpressing the RecA and CheW mutants were transformed to *S. enterica* wild-type cells, and cultured on swarming plates containing 30 μM of IPTG. In all cases, it was confirmed by ELISA that the RecA and CheW concentration increases were at least more than 20-fold for RecA and 100-fold for CheW after IPTG induction. Representative images of *in vivo* swarming assays of *S. enterica* wild-type strains overexpressing RecA and CheW mutant derivatives that allow or impair RecA-CheW interaction are shown in **Figure 5B**. The results for all mutant derivatives are summarized in **Tables 2** and **3**. In agreement with the data obtained in the *in vitro* co-immunoprecipitation assays, only the mutant derivatives unable to interact with the corresponding wild-type protein do not inhibit swarming when overexpressed. These results confirm the importance of these residues in RecA-CheW *in vivo* interaction. Further, it is worth noting that none of the non-interacting RecA or CheW mutant derivatives are able to recover the swarming ability of the either *S. enterica ΔrecA* or *ΔcheW* strains (data not shown).

**FIGURE 6:**
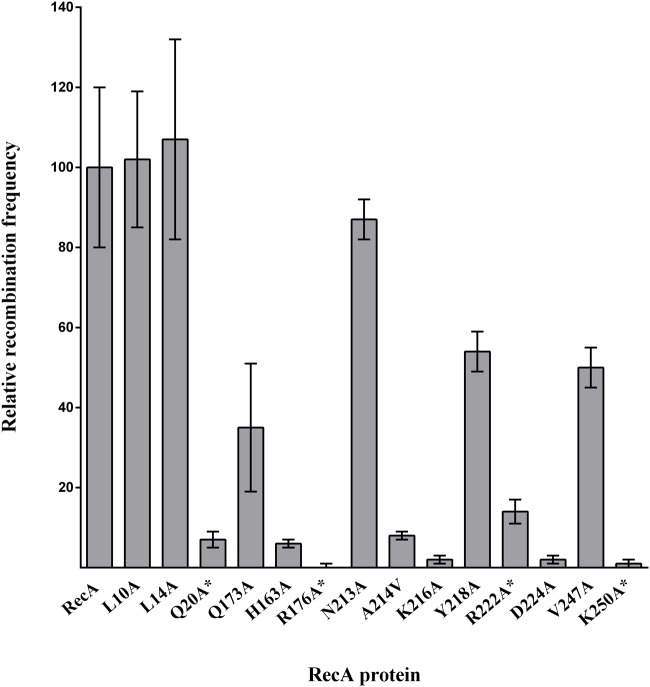
***In vivo* recombination activity of the RecA mutant derivatives.** The efficiency of strains, containing the overexpression vector carrying the corresponding RecA derivative, to recombine the selectable genetic marker transduced by bacteriophage P22intH7 was tested. The relative recombination frequency was calculated as the recombination efficiency of each mutant derivative with respect to that of the strain overexpressing wt RecA, and the recombination efficiency of each strain as the number of transductants compared to the initial recipient cell concentration. The RecA mutant derivatives unable to interact with CheW are indicated by an asterisk (^∗^). The relative recombination frequencies were calculated as the mean of three independent experiments. Error bars indicate the standard deviation.

The three domains of RecA exhibit functional overlap (Takahashi et al., 1996; McGrew and Knight, 2003; Adikesavan et al., 2011). For example, in *E. coli*, Arg176 and Lys250 RecA residues, identified in this work as essential for the RecA-CheW interaction, are involved in recombination activity (Chen et al., 2008; Adikesavan et al., 2011). To determine whether the interaction interfaces associated with RecA-CheW coupling also have other overlapping functions, the recombination ability of the obtained RecA derivatives was determined. The results showed that all of the RecA mutants causing impaired RecA-CheW coupling had also lost their recombination ability (**Figure 6**). Further, some other residues that are not involved in RecA-CheW pair formation (H163A, A214V, K216A, and D224A) also present a clear decrease in their recombinase activity (**Figure 6**). These data are not surprising since as stated above, their location matches with regions previously described to be associated with recombination (Chen et al., 2008; Adikesavan et al., 2011).

### CheW Subcellular Location in a Cell Unable to Form RecA-CheW Pair

Once identified the RecA and CheW residues associated with interaction and to unequivocally associate the RecA-CheW pair formation with CheW location, the behavior of the non-CheW-interacting RecA R176A mutant was analyzed. In this case, the *recA* mutant derivative (R176A) was overexpressed in a *S. enterica ΔrecA* strain under non-DNA-damaging conditions and the locations of the CheW and RecA tagged proteins were examined.

**FIGURE 7:**
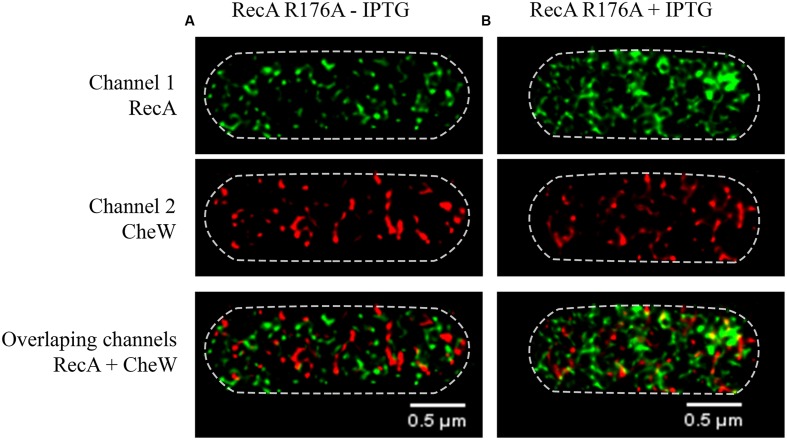
**Representative STED images of the subcellular locations of RecA and CheW in the *S. enterica ΔrecA* strain overexpressing the non-CheW-binding RecA R176A mutant.** **(A)** The effect of basal expression, in the absence of IPTG, of RecA R176A mutant on RecA-CheW distribution is shown. **(B)** Addition of the inducer (30 μM of IPTG) yielded the overexpression of the non-CheW-binding RecA R176A mutant. RecA and CheW proteins were labeled with Alexa Fluor^®^ A488 (channel 1) and Alexa Fluor^®^ A568 (channel 2), respectively. In the images, each channel is shown individually and overlapped. In all cases, the maximum intensity projection images of the obtained z-stacks are shown.

As it is seen in **Figure 7**, the overexpression of the RecA R176A mutant by IPTG addition does not prompt any change in CheW distribution, as it happens when wild-type RecA is overexpressed (**Figure 2**). In the presence of RecA R176A mutant protein the CheW was never located at cell poles regardless the RecA concentration (**Figure 7**).

These results indicate that not only the concentration of RecA but also the ability of the protein to interact with CheW is required for CheW distribution and thus for chemoreceptor clustering at the cell poles, *a sine qua non* condition for bacterial colony swarming.

## Discussion

The experiments performed herein have identified the protein interfaces involved in the interaction between RecA and CheW in *S. enterica*. The regions of CheW specifically associated with RecA are β1, β4, T6, and B6 (**Figure 1**; **Table 3**), which are not those that interact with CheA, CheW, or MPCs (Bilwes et al., 1999; Underbakke et al., 2011; Cassidy et al., 2015). Accordingly, the interaction of RecA and CheW should not interfere with any of the three CheW-binding targets identified thus far (CheA, CheW, and MCPs). The interaction interfaces of RecA are located within the N-terminal and central domains, thus involving the α1, α10, and β8 regions of the protein (**Figure 3**; **Table 2**). These are the same regions previously reported to be involved in RecA polymer formation, ATP hydrolysis, and ssDNA and LexA interactions (Story et al., 1992; Campbell and Davis, 1999; Chen et al., 2008; Adikesavan et al., 2011), such that none of the non-CheW interacting RecA derivatives here described were able to carry out recombination (**Figure 6**). These results suggest that when a molecule of RecA is interacting with CheW, it cannot participate in DNA recombination and repair. Nevertheless, only part of the total RecA amount present in a SOS-induced cell will be associated to CheW hijack, ensuring that DNA repair and recombination take place in the DNA-damaged cells.

By using super-resolution 3D-STED, we were able to show that following SOS induction the increase in the concentration of RecA, but not the activation of other SOS response associated functions, is enough to induce the redistribution of CheW from aggregates at the cell poles to foci with a helicoid configuration along the cell axis, showing the same subcellular location than RecA (**Figures 1B** and **2B**). This finding is consistent with the impairment of the chemoreceptor array assembly that occurs when the SOS response is activated (Irazoki et al., 2016). By contrast, in cells carrying a non-CheW-interacting mutant RecA, CheW is unable to cluster at the cell poles (**Figure 7**). Thus pointing out that the interaction between RecA and CheW is essential for both swarming modulation (**Figure 5**) and CheW clustering at the cell poles (**Figure 7**), and confirming the previously described for *S. enterica* RecA-defective strains, in which chemoreceptor array assembly was inhibited (Mayola et al., 2014).

**FIGURE 8:**
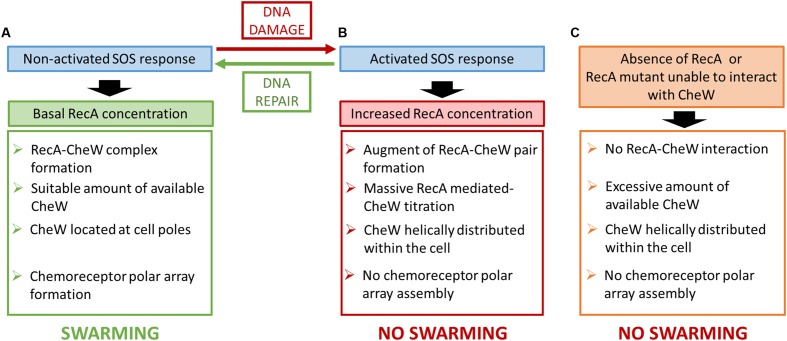
**Schematic diagram of the putative mechanism for the control of *S. enterica* swarming motility by RecA and SOS response.** The effect of non-activated SOS response **(A)**, the induction of the SOS system **(B)** and the absence or the presence of a non-interacting RecA mutant **(C)** is proposed.

Taken together, these results suggest two different scenarios to explain the role of RecA in chemoreceptor polar cluster formation and swarming modulation. Thus, RecA may be a component of the chemoreceptor array, since either its absence or overexpression interferes directly with chemoreceptor assembly. However, this is unlikely since polar array clusters have been characterized in detail (Li et al., 2013; Briegel et al., 2014b; Cassidy et al., 2015; Eismann and Endres, 2015) and their well-organized structure does not seem to allow for RecA attachment. Alternatively, RecA could prompt the titration of CheW, thus preventing chemoreceptor assembly and therefore also polar cluster array formation during activation of the SOS response (**Figure 8**). A similar control strategy has been described for other interacting proteins whose regulatory function relies on the availability of the protein with which they interact (Liu and Richardson, 1993; Plumbridge, 2002; Hill et al., 2013; Hernández-Rocamora et al., 2015; Paget, 2015).

The concentration of CheW is essential for chemoreceptor cluster formation and the absence or overexpression of this protein inhibits array assembly (Avram Sanders et al., 1989; Cardozo et al., 2010). In addition, a recent study showed that in these arrays, CheW not only serves as an adaptor protein anchoring MCPs to CheA but that, via ring formation, it is also responsible for chemoreceptor array stability (Cassidy et al., 2015). Therefore, in the absence of DNA damage, RecA is able to bind CheW adjusting the availability of this protein needed to allow chemosensory system assembly and thus swarming ability (**Figure 2A**).

Since activation of the SOS response increases the concentration of RecA but not of CheW (Irazoki et al., 2016), then during SOS activation the amount of RecA-CheW complex formation will be stimulated but the CheW availability will thereby be reduced (**Figure 1A**), which will affect the stability of the hexagonal receptor signaling array (Cassidy et al., 2015). Following DNA damage repair, the *recA* expression returns to its basal level restoring chemoreceptor array assembly and thus swarming ability (Irazoki et al., 2016) returning the cell the non-DNA damage condition (**Figure 1B**). This explains why only CheW overexpression can reestablish polar cluster assembly in a RecA-overexpressing strain (Irazoki et al., 2016), i.e., the increased availability of CheW restores chemosensory array assembly. Moreover, the absence of RecA (Mayola et al., 2014) or an inability of the protein to interact with CheW (**Figure 7**) will increase the available amount of CheW (**Figure 7A**), thus interfering with chemoreceptor ring structuring and cluster formation (Cardozo et al., 2010).

Besides CheW is a key protein in the *S. enterica* chemoreceptor pathway (Baker et al., 2006) and RecA seem to play a role in chemotaxis (Mayola et al., 2014), chemoreceptor arrays are not essential for chemotaxis response. It has been previously reported that despite the absence of structuration of polar clusters, the association of chemoreceptors with the chemotaxis pathway is still functional (Maki et al., 2000; Briegel et al., 2014b). Furthermore, chemotaxis and swimming are not affected when *E. coli* is treated with cephalexin (Maki et al., 2000), a beta-lactam antibiotic that induce SOS response (Bano et al., 2014). Moreover, biofilm formation is affected neither by the absence or the overexpression of RecA (data not shown).

It is also worth noting that although the absence or overexpression of RecA inhibits cluster assembly, the presence of chemoreceptor clusters is not completely abolished in either *ΔrecA* or RecA overexpressing strains. It has been widely reported that chemoreceptor arrays are highly stable structures. Several models have been proposed to describe its assembly and stabilization and not only CheW but also CheA and even the cell membrane curvature seem to be involved (Shiomi et al., 2005; Thiem and Sourjik, 2008; Greenfield et al., 2009; Jones and Armitage, 2015). Then, the alteration in CheW availability would clearly affect chemoreceptor cluster assembly but not completely abolish it. Accordingly, once the SOS response is activated, more than the 70% of the cells presented CheW distributed along the cell instead of being at cell poles due to the RecA mediated – CheW titration.

As mentioned in Section “Introduction” RecA is associated to the inner-membrane anionic phospholipids (Rajendram et al., 2015). This interaction is necessary for RecA activity during DNA damage repair. However, the RecA residues that interact with anionic phospholipids do not overlap with those interacting with the CheW interface (**Table 2**). RecA proteins also form foci that may or may not be associated with DNA (Renzette et al., 2005). The DNA-less proteins, referred to as RecA storage structures, are often located at the cell poles and are redistributed along the cell in response to DNA damage (Renzette et al., 2005; Lesterlin et al., 2014; Rajendram et al., 2015). Interestingly, an *E. coli* R28A RecA mutant with an amino acid substitution in the α1 RecA region, shown in this study to be associated with RecA-CheW pair formation in *S. enterica* (**Table 2**), is unable to generate DNA-less RecA foci (Renzette and Sandler, 2008). Thus, our results suggest that in addition to RecA storage, DNA-less RecA foci participate in the modulation of swarming motility.

Our data therefore shed light on a new role of RecA, the titration effect on CheW protein, which based on protein–protein interaction strategy, modulates the CheW distribution within the cell thus controlling the swarming ability.

## Author Contributions

OI, JA, and SC performed the *in silico* analyses and site-directed mutagenesis. OI and SC analyzed swarming phenotypes and chemoreceptor polar cluster assembly. OI, SC, and TZ performed the fluorescent immunolabeling and STED imaging. OI, SC, and JB conceived the experiment, coordinated the research, discussed the findings and interpreted the results.

## Conflict of Interest Statement

The authors declare that the research was conducted in the absence of any commercial or financial relationships that could be construed as a potential conflict of interest.
