# Supplementary material for: Molecular Interaction and Cellular Location of RecA and CheW Proteins in Salmonella enterica during SOS Response and Their Implication in Swarming
Source: Front Microbiol. 2016 Oct 6;7:1560. doi: 10.3389/fmicb.2016.01560 (PMC5052270; doi:10.3389/fmicb.2016.01560)
Supplement: Supplementary file 1 [file Table_1.PDF]

Supplementary Material

Molecular interaction and cellular location of RecA and CheW proteins in *Salmonella enterica* during SOS response and their implication in swarming

Oihane Irazoki, Jesús Aranda, Timo Zimmermann, Susana Campoy*, Jordi Barbé.

*** Correspondence:** Susana Campoy: Susana.campoy@uab.cat

Table S1. Oligonucleotides used in this work

Name	Sequence (5'→ 3')	Application
**recA_NdeI**	ggaattccatatgGCTATCGACGAAAACAAACAG	Oligonucleotides for *recA::HA* fusion construction
**recA1xHA_BamHI**	cgggatccTTA*AGCGTAATCTGGAACATCGTATGGGTA*gccgccgccAAAATCTTCGTTGGTTTCTG	
**recA-L10V_NdeI_F**	ggaattccatatgATGGCTATCGACGAAAACAAACAGAAAGCGGCGGCGGCAGCACTG	Oligonucleotides for RecA L10V mutant derivative construction
**recA-L14V_NdeI_F**	ggaattccatatgATGGCTATCGACGAAAACAAACAGAAAGCGTTGGCGGCAGCAGCGGGCCAAATTGAAAAGCAATTTGGT	Oligonucleotides for RecA L14V mutant derivative construction
**recA-Q20E_NdeI_F**	ggaattccatatgATGGCTATCGACGAAAACAAACAGAAAGCGTTGGCGGCAGCACTGGGCCAAATTGAAAAGGCATTTGGTAAAGGCGAAGACCGTTCT	Oligonucleotides for RecA Q20E mutant derivative construction
**recA-H163A_F**	CGGAAATCGAAGGCGAAATCGGCGACTCTGCCATGGGCCTCGCGGCGCGTATGATGAGCCAGGC	Oligonucleotides for RecA H163A mutant derivative construction
**recA-H163A_R**	GCCTGGCTCATCATACGCGCCGCGAGGCCCATGGCAGAGTCGCCGATTTCGCCTTCGATTTCCG	
**recA-Q173A_F**	GCCTCGCGGCGCGTATGATGAGCGCGGCGATGCGTAAGCTGGCGGGGAACCTGAAACAGTCC	Oligonucleotides for RecA Q173A mutant derivative construction
**recA-Q173A_R**	GGACTGTTTCAGGTTCCCCGCCAGCTTACGCATCGCCGCGCTCATCATACGCGCCGCGAGGC	

**recA-R176A_F**	GCCTCGCGGCGCGTATGATGAGCCAGGCGATGGCTAAGCTGGCGGGGAACCTGAAACAGTCC	Oligonucleotides for RecA R176A mutant derivative construction
**recA-R176A_R**	GGACTGTTTCAGGTTCCCCGCCAGCTTAGCCATCGCCTGGCTCATCATACGCGCCGCGAGGC	
**recA-N213A_F**	CGGTAACCCGGAAACCACCACCGGCGGTGCCGCGCTGAAATTCTACGCCTCTGTTCGTCTTG	Oligonucleotides for RecA N213A mutant derivative construction
**recA-N213A_R**	CAAGACGAACAGAGGCGTAGAATTTCAGCGCGGCACCGCCGGTGGTGGTTTCCGGGTTACCG	
**recA-A214V_F**	CGGTAACCCGGAAACCACCACCGGCGGTAACGTGCTGAAATTCTACGCCTCTGTTCGTCTTG	Oligonucleotides for RecA A214V mutant derivative construction
**recA-A214V_R**	CAAGACGAACAGAGGCGTAGAATTTCAGCACGTTACCGCCGGTGGTGGTTTCCGGGTTACCG	
**recA-K216A_F**	CGGAAACCACCACCGGCGGTAACGCGCTGGTATTCTACGCCTCTGTTCGTCTTGATATCC	Oligonucleotides for RecA K216A mutant derivative construction
**recA-K216A_R**	GGATATCAAGACGAACAGAGGCGTAGAATACCAGCGCGTTACCGCCGGTGGTGGTTTCCG	

**recA-Y218A_F**	**CCACCACCGGCGGTAACGCGCTGAAATTCGCCGCCTCTGTTCGTCTTGATATCC GTCGTATTGG**	Oligonucleotides for RecA Y218A mutant derivative construction
**recA-Y218A_R**	CCAATACGACGGATATCAAGACGAACAGAGGCGGCGAATTTCAGCGCGTTACCGCC GGTGGTGG	
**recA-R222A_F**	GGTAACGCGCTGAAATTCTACGCCTCTGTTGCTCTTGATATCCGTCGTATTGGCGCGG	Oligonucleotides for RecA R222A mutant derivative construction
**recA-R222A_R**	CCGCGCCAATACGACGGATATCAAGAGCAACAGAGGCGTAGAATTTCAGCGCGTTA CC	
**recA-D224A_F**	GCTGAAATTCTACGCCTCTGTTCGTCTTGCTATCCGTCGTATTGGCGCGGTGAAAGAG GGC	Oligonucleotides for RecA D224A mutant derivative construction
**recA-D224A_R**	GCCCTCTTTCACCGCGCCAATACGACGGATAGCAAGACGAACAGAGGCGTAGAATTT CAGC	
**recA-V247A_F**	CGTGGGTAGCGAAACGCGTGTGAAAGTGGCGAAAAACAAAATCGCCGCGCCGTTTA AGC	Oligonucleotides for RecA V247A mutant derivative construction
**recA-K250A_F**	CGAAACGCGTGTGAAAGTGGTGAAAAACGCAATCGCCGCGCCGTTTAAGCAGGCCG AGTTCC	Oligonucleotides for RecA K250A mutant derivative construction
**recA-K250A_R**	GGAACTCGGCCTGCTTAAACGGCGCGGCGATTGCGTTTTTCACCACTTTCACACGCGT TTCG	

**cheW-NdeI**	**ggaattccatatgACCGGTATGAGTAATGTAAGC**	Oligonucleotides for *cheW::FLAG* fusión construction
**che1xFLAG_BamHI**	cgggatccTTA***TTTGTCGTCGTCGTCTTTGTAGTC****gccgccgcc*CGCGACGTGTGATGCTGCGATATCC	
**cheW-F21A_F**	catatgATGACCGGTATGAGTAATGTAAGCAAACTGGCCGGCGAGCCGTCAGGTCAGGAATTCCTGGTGGCUACACTGGGAAATGAAGAGTACGGCATCG	Oligonucleotides for CheW F21A mutant derivative construction
**cheW-K55A_F**	GCGCATCGCCAATACACCGGCCTTTATAGCAGGGGTGACTAACCTGCGCGGCGTGATTGTCCC	Oligonucleotides for CheW K55A mutant derivative construction
**cheW-K55A_R**	GGGACAATCACGCCGCGCAGGTTAGTCACCCCTGCTATAAAGGCCGGTGTATTGGCGATGCGC	
**cheW-D83A_F**	GGCGACGTTGAGTACGATGCCAATACGGTAGTGATCGTAC	Oligonucleotides for CheW D83A mutant derivative construction
**cheW-D83A_R**	GTACGATCACTACCGTATTGGCATCGTACTCAACGTCGCC	
**cheW-S109A_F**	GGGATAGTGGTAGACGGCGTGTCTGACGTAGCGTCGTTAACGGCGGAACAGATCCGTCCGGCG	Oligonucleotides for CheW S109A mutant derivative construction
**cheW-S109A_R**	CGCCGGACGGATCTGTTCCGCCGTTAACGACGCTACGTCAGACACGCCGTCTACCACTATCCC	

**cheW-F121A_F**	**GGAACAGATCCGTCCGGCGCCAGAAGCTGCCGTGACCTTGTCAACAGAATATTTGACG**	Oligonucleotides for CheW F121A mutant derivative construction
**cheW-F121A_R**	CGTCAAATATTCTGTTGACAAGGTCACGGCAGCTTCTGGCGCCGGACGGATCTGTTCC	
**recA_BamHI**	ggaattggatccGCTATCGACGAAAACAAACAG	Oligonucleotide used together with recA1xHA_BamHI primer for recA cloning into pKO3 vector
**recAP1**	GCGACCGTGAATCGGTGCGTCGACAGGCGACGACATACGCGCTACAAACCCTGTGGCAACAATTTCTACAAAACACTTGAGTGTAGGCTGGAGCTGCTTC	Oligonucleotides for the Δ*recA* strain construction by one step inactivation
**recAP2**	ACTCCTGTCATGCAACTTGGTATTGAACCGGATAGTGAATTCGTACTGTTGAAGCAATTATATTGTATGCTCATACATTAATGGGAATTAGCCATGGTCC	
**recAext_f**	CAGTGGAGAAGGGATTACGC	
**recX_r**	TAATTCCCATTCATCAGGTGATGGTGGG	

[a] underlined nucleotides correspond to restriction enzymes, upper-case italics correspond to HA-tag sequence, bold upper-case italics to the FLAG-tag sequence and lower-case underlined ones to the glycine-linker.
